# Contraception free of charge for young women in Tuscany: an interrupted time series analysis

**DOI:** 10.1093/eurpub/ckac129.390

**Published:** 2022-10-25

**Authors:** A Ferrari, M Bonciani, I Corazza, C Tortù, M Vainieri

**Affiliations:** Management and Health Laboratory, Sant'Anna School of Advanced Studies, Pisa, Italy

## Abstract

The Regional Health Authority of Tuscany, ITALY, launched in November 2018 a public program providing all contraceptive methods free of charge to all young residing women after medical evaluation at free family counselling centres. We aimed to explore the effect of the regional program on five outcomes - abortions, contraception provision, access to family counselling centres, conception, and outpatient service utilization for six sexually transmitted diseases (STDs). These outcomes were retrospectively computed from January 2016 to December 2020 using regional administrative databases, and then expressed as both annual rates per 1,000 and monthly rates per 100,000 women aged 14 to 25 years and residing in Tuscany. We ran interrupted time series (ITS) models to analyze how the trends in monthly rates changed over time after the intervention (set in November 2018). The decline in annual abortion rates per 1,000 women doubled from 2016-2018 (-9%, from 7.0 to 6.4) to 2018-2020 (-17%, from 6.4 to 5.3). ITS models revealed a significant post-intervention decrease in monthly abortion rates (-0.61, -1.11 to -0.12) and a significant increase in both access rates to counselling centres (87.60, 77.77 to 97.43) and contraception provision rates (16.75, 0.31 to 33.19). Conception rates fell significantly just in under-18 women (-0.84, -1.39 to -0.28). A significant reduction in outpatient service utilization rates for STDs emerged for N. gonorrhoeae, HSV, and HPV. As a prime example of similar initiatives in Italy, the free contraception program of Tuscany promoted access to counselling and contraception services among young under-25 women, empowering the entire care pathway. As a result, abortion rates declined significantly in this group. The wider access to counselling centres to obtain free contraception and overall enhancement of the pathway might have fostered sexual education and reproductive health, leading to a reduction in teenage conception and STD spread.

**Key messages:**

• This study proves the effectiveness of the free contraception campaign of Tuscany in reducing abortions and empowering the entire care pathway, providing further evidence for policy accountability.

• Therefore, this study supports the maintenance of this program and suggests the potential benefits of implementing and promoting similar initiatives in other Regions.

